# A novel dual-site OFC-dlPFC accelerated repetitive transcranial magnetic stimulation for depression: a pilot randomized controlled study

**DOI:** 10.1017/S0033291724002289

**Published:** 2024-10

**Authors:** Hailun Cui, Hui Ding, Lingyan Hu, Yijie Zhao, Yanping Shu, Valerie Voon

**Affiliations:** 1 Department of Psychiatry, University of Cambridge, Cambridge, UK; 2 Institute of Science and Technology for Brain-Inspired Intelligence, Fudan University, Shanghai, China; 3 Department of Neurosurgery, Huashan Hospital, Shanghai Medical College, Fudan University, Shanghai, China; 4 Department of Radiology, The Second People's Hospital of Guizhou Province, Guiyang, China; 5 Department of Psychiatric Rehabilitation, The Second People's Hospital of Guizhou Province, Guiyang, China; 6 Department of Psychiatry of Women and Children, The Second People's Hospital of Guizhou Province, Guiyang, China; 7 Key Laboratory of Computational Neuroscience and Brain-Inspired Intelligence (Fudan University), Ministry of Education, China; 8 Zhangjiang Fudan International Innovation Centre, Shanghai, China

**Keywords:** Accelerated, fMRI, Orbitofrontal cortex, Subcallosal cingulate, Transcranial magnetic stimulation, Treatment resistant depression

## Abstract

**Background:**

This study aimed to evaluate a novel rTMS protocol for treatment-resistant depression (TRD), using an EEG 10–20 system guided dual-target accelerated approach of right lateral orbitofrontal cortex (lOFC) inhibition followed by left dorsolateral prefrontal cortex (dlPFC) excitation, along with comparing 20 Hz dlPFC accelerated TMS *v.* sham.

**Methods:**

Seventy five patients participated in this trial consisting of 20 sessions over 5 consecutive days comparing dual-site (cTBS of right lOFC followed sequentially by 20 Hz rTMS of left dlPFC), active control (sham right lOFC followed by 20 Hz rTMS of left dlPFC) and sham control (sham for both targets). Resting-state fMRI was acquired prior to and following treatment.

**Results:**

Hamilton Rating Scale for Depression (HRSD-24) scores were similarly significantly improved at 4 weeks in both the Dual and Single group relative to Sham. Planned comparisons immediately after treatment highlighted greater HRSD-24 clinical responders (Dual: 47.8% v. Single:18.2% v. Sham:4.3%, *χ*2 = 13.0, *p* = 0.002) and in PHQ-9 scores by day 5 in the Dual relative to Sham group. We further showed that accelerated 20 Hz stimulation targeting the left dlPFC (active control) is significantly better than sham at 4 weeks. Dual stimulation decreased lOFC-subcallosal cingulate functional connectivity. Greater baseline lOFC-thalamic connectivity predicted better therapeutic response, while decreased lOFC-thalamic connectivity correlated with better response.

**Conclusions:**

Our novel accelerated dual TMS protocol shows rapid clinically relevant antidepressant efficacy which may be related to state-modulation. This study has implications for community-based accessible TMS without neuronavigation and rapid onset targeting suicidal ideation and accelerated discharge from hospital.

## Introduction

Major depressive disorder (MDD) is a significant public health concern and ranks as a leading global cause of disability (Disease, Injury, & Prevalence, 2018). Patients with treatment-resistant depression (TRD) experience higher mortality rates and bear a greater economic burden (Li et al. 2019; Sussman, O'Sullivan, Shah, Olfson, & Menzin, 2019). Standard repetitive transcranial magnetic stimulation (rTMS) protocols delivering high frequency (HF) stimulation daily over the left dorsolateral prefrontal cortex (dlPFC) over 20 sessions show considerable clinical improvements (Hyde et al. 2022; O'Reardon et al. 2007). However, given its limited capacity for treating patients with time restraints or with acute severity or suicidality, there is room for expediting treatment duration and optimizing the target selection (Sonmez et al. 2019).

Accelerated TMS (aTMS), an emerging treatment delivery schedule, shortens the duration of the treatment course from weeks to days by increasing the number of daily sessions from one to multiple. This intensified rTMS protocol has since been extensively examined clinically and modified to test both HF and theta burst stimulation protocols (Baeken et al. 2013; Duprat et al. 2016), resulting in a reduction of the treatment duration by at least two times, while retaining overall safety and efficacy (Caulfield, Fleischmann, George, & McTeague, 2022; Sonmez et al. 2019). Following a strong remission rate in severely depressed patients, where 11 out of 14 participants (79%) satisfied the remission criteria in a placebo-controlled randomized controlled trial with no major side effects, the FDA has lately cleared the application of the Stanford neuromodulation therapy (SNT) in the treatment of MDD (Cole et al. 2022; Cole et al. 2020). In addition to a large antidepressant effect, some have suggested a potential for rapid response for accelerated protocols (Desmyter et al. 2016; George et al. 2014), as the expected onset of therapeutic effects is typically correlated with the frequency or speed at which the interventions are administered (Chen et al. 2023). While consensus is lacking across studies at present, it is important to explore aTMS protocols due to the scarcity of alternative therapies, with electroconvulsive therapy being the only alternative that provide both rapid antidepressant effects and comparable safety profiles to TMS treatment in the present management of TRD (Chen et al. 2023).

The presence of varying response rates and trajectories indicates that different neurobiological patterns may be responsible for higher levels of responsiveness to neurostimulation methods. The complexity underlying depression network circuitry suggest potential heterogenous pathology implicating different key nodes, which drives the search for new treatment strategies including novel cortical targets (Drysdale et al. 2017). The orbitofrontal cortex (OFC), for instance, has emerged as a promising site for neuromodulation. The OFC is primarily implicated in the pathophysiology of OCD, exhibiting heightened resting-state functional connectivity (FC) with the striatum (Cocchi et al. 2018; Harrison et al. 2009), thereby representing a major prefrontal constituent of the cortico-striato-thalamo-cortical (CSTC) circuits that underlie OCD symptomatology (Milad & Rauch, 2012). TMS treatments targeting OFC and neighboring prefrontal regions (e.g. the frontal pole area), especially protocols implementing low frequency (LF) stimulations to mitigate the functional hyperactivity in this region, have shown largely positive outcomes in alleviating OCD symptoms (Kumar, Singh, Chadda, Verma, & Kumar, 2018; Nauczyciel et al. 2014; Ruffini et al. 2009), albeit with inconsistent outcomes (Cocchi et al. 2023; Dutta et al. 2021). In depression, with its reported role in punishment/non-reward signaling (Rolls, 2016; Rolls, Cheng, & Feng, 2020), OFC has been considered as an important alternative to the left dlPFC (Cash et al. 2021), with pilot open-label studies of LF rTMS of the right lateral OFC showing potential efficacy for depression (Feffer et al. 2018; Rao et al. 2018). Unlike the FC between the dlPFC and subgenual cingulate cortex (SCC), the OFC has a direct anatomical connection to the SCC (Burks et al. 2018; Garcia-Cabezas & Barbas, 2017), with an elevated FC strength observed in depression possibly representing increased impact from the OFC to the SCC related to unpleasant, non-reward stimuli (Rolls et al. 2019).

Thus, we tested the clinical efficacy in TRD patients with a three-arm double-blind randomized controlled trial implementing an accelerated novel dual-site stimulation protocol (putative inhibitory TMS (continuous theta burst stimulation, cTBS) targeting right lateral OFC followed by putative excitatory TMS (20 Hz rTMS) targeting left dlPFC), comparing with an active single-site control (sham OFC followed by left dlPFC TMS) and sham (sham OFC and sham dlPFC). We hypothesize that stimulating two distinct cortical networks sequentially in an accelerated manner may enhance anti-depressant effects of TMS. We also conducted pre- and post-resting functional MRI investigating treatment-associated FC changes, hypothesizing a decrease in functional coupling between target OFC and SCC following cTBS, and a modulatory effect of HF rTMS between target dlPFC and medial prefrontal cortex (mPFC) (Liston et al. 2014). We also conducted analyses to identify longitudinal FC correlates of treatment response and potential response predictors.

## Methods and materials

This assessor- and patient-blinded, single-center randomized controlled clinical trial was registered with ChiCTR (Identifier: ChiCTR2100049002) and was conducted from June 2021 to February 2022, with follow-up completed by April 2022. Participants were recruited from the Second People's Hospital of Guizhou Province (Guizhou Mental Health Centre). Power analysis targeted 80% power (*α* = 0.05) to detect a medium effect (Cohen’ *f* = 0.18) on interventional effect at the primary end point (see online Supplement for further details). Participants provided written informed consent as approved by the Institutional Review Board of Guizhou Mental Health Centre (Ethics identifier: [2021]58).

### Participants

Participants were recruited by referral from psychiatrists. Eligibility criteria were men and women aged 18–55 years with a primary diagnosis of MDD (single or recurrent episode) based on DSM-V criteria, and a score ⩾21 on 24-item Hamilton Rating Scale of Depression (HRSD-24), with no previous TMS treatment experience. Duration of the current depressive episode was less than 5 years with treatment resistance based on lack of clinical response or intolerance to at least 2 but not more than 4 antidepressant treatments of adequate dose and duration in the current episode. By limiting the number of previous failed trials and duration, we ensured we accurately represented failed trials by corroborating clinical notes. Patients were required to be stable on medication for at least 6 weeks prior to study entry with no change in medication throughout the study. The inclusion and exclusion criteria are presented in the supplement materials.

### Randomization and blinding of treatment

**Figure 1.**
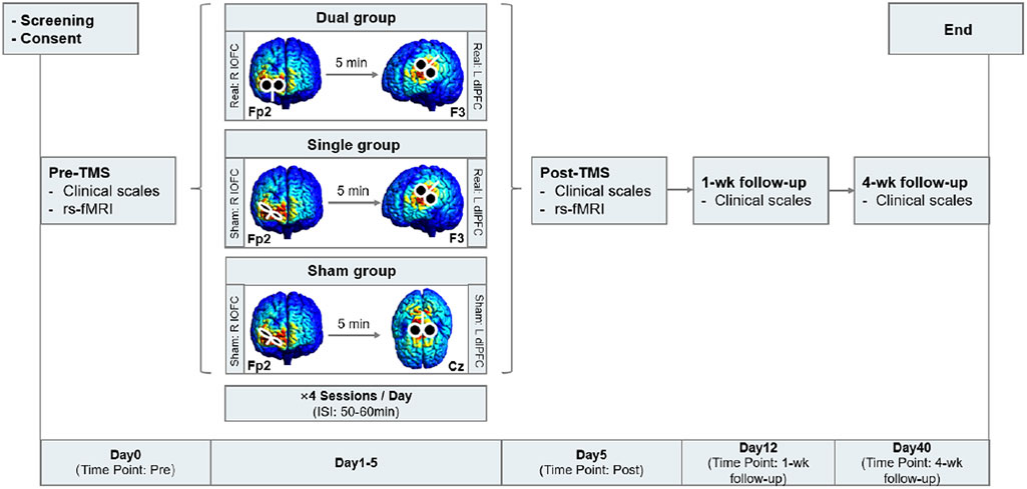
Coil position and study procedure. An illustration of the coil position and stimulus protocol is provided. Two cortical targets were involved, including the right lateral orbitofrontal cortex (R lOFC) using the EEG-Fp2 position and the left dorsolateral prefrontal cortex (L dlPFC) using the EEG-F3 position. The sham stimulation for the R lOFC target was delivered with the coil tilted 90 degrees, such that the edge of the coil touched the scalp. The sham stimulation for the L dlPFC target was delivered to the vertex area (EEG-Cz). We sought the coil position and orientation that generated the electric field magnitude at the cortical target and visualized the results using SimNIBS 3.2, a free software for electric field modelling in non-invasive brain stimulation including TMS, with rapid coil placement optimization facilitated through an auxiliary dipole method (ADM) (Gomez, Dannhauer, & Peterchev, 2021). The resulting electric field maps were generated using the figure-of-8 coil and was subsequently converted into MNI-space. Abbreviations: rs-fMRI, resting state functional MRI; ISI, intersession interval.

Using computer-generated randomization sequences, patients were 1:1:1 assigned to receive 1 of the 3 following treatments: (i) active cTBS targeting right lateral OFC followed by active 20 Hz rTMS targeting left dlPFC (Dual group); (ii) sham cTBS right lateral OFC followed by active 20 Hz rTMS left dlPFC (Single group), and (iii) sham cTBS right lateral OFC followed by sham rTMS left dlPFC (Sham group). Participants were TMS-naïve with no previous non-pharmacological treatments. The assessor and patients were blinded to the TMS conditions. A graphic overview of the study is presented in Fig. 1. The efficacy of blinding was systematically assessed (see online supplementary materials).

### Transcranial magnetic stimulation

A total of 20 sessions (a single session: right lateral OFC followed by left dlPFC, separated by a 5 min interval) of TMS were administered for 5 consecutive days with 4 sessions per day using CCY-I TMS instrument (Yiruide Co., Wuhan, China) and a ‘figure-of-8’ coil (Shen, Cao, Shan, Dai, & Yuan, 2017; Yu et al. 2020). Sham TMS was carried out with the coil tilted 90° perpendicular to the skull for the right lateral OFC, and over the interhemispheric fissure at the vertex for the left DLPFC, with pulses delivered at a low intensity (10% resting motor threshold, rMT) to elicit similar skin sensations as real stimulation. Further details regarding the rationale for the sham procedures is described in the supplementary materials. The participant's rMT was determined as the minimum stimulus required to induce right thumb contraction ⩾5/10 times. The standardized international 10–20 EEG system was used to position the TMS coil: Fp2 (10% dorsal from the nasion to inion, 10% lateral) for the right lateral OFC, and F3 for left dlPFC (Fig. 1). cTBS over the right lateral OFC consisted of 600 pulses per session delivered as triplets at 50 Hz repeated at 5 Hz at 70%–100% rMT on the first two sessions based on personal level of tolerance, and scaled up to 100% starting from the third session. HF rTMS over the left dlPFC consisted of ninety trains of 1s stimuli with 10s inter-train intervals administered at a frequency of 20 Hz and an intensity of 100% of the individual's rMT, providing 1800 pulses per session. A single session involving OFC followed by dlPFC with a 5-min interval lasted about 22 min. Intersession intervals (ISI) were 50 to 60 min. Altogether, each participant received 12 000 pulses over right lateral OFC, and 36 000 pulses over left dlPFC.

### Outcomes

The primary outcome measure was the absolute change of HRSD-24 total score (0–7 indicates no depression; 8–19, mild; 20–34, moderate; and 35 severe depression) from baseline (time point: Pre) to post-intervention (time point: Post, 1-week and 4-week follow-up). Further secondary endpoints included rates of response (percent reduction of at least 50% from baseline) and remission (score <8) based on HRSD-24, the clinician-administered Hamilton Rating Scale of Anxiety (HAMA, score range: 0–56) (Hamilton, 1959) assessed from baseline to post-intervention, and the 9-question Patient Health Questionnaire (PHQ-9, score range: 0–27) (Kroenke & Spitzer, 2002) assessed from day 1 to 5 after daily treatment sessions.

### MRI data acquisition and analysis

All patients underwent resting state functional MRI scanning at baseline and after intervention (time point: Post, collected within 120 min after completing the final TMS treatment session) to investigate the effects of TMS on FC with two target areas (right lateral OFC and left dlPFC) as regions of interest (ROIs). Functional images were preprocessed with default settings and denoised using aCompCor using the CONN toolbox v20.b (Whitfield-Gabrieli & Nieto-Castanon, 2012). Further details of data acquisition and preprocessing steps are reported in the supplementary materials.

The FC analyses were carried out using the CONN toolbox. To investigate FC differences across three groups of participants, two target areas were defined as ROIs: left dlPFC (Montreal Neurological Institute [MNI] coordinates = −38, 25, 48; 10-mm radius sphere), corresponding to the EEG positions F3 of the international 10–20 electrode system chosen as the stimulation site of left dlPFC (Herwig et al. 2003), and right anterior and lateral orbitofrontal cortex (lOFC), created following AAL3 map labels using WFU Pickatlas software, corresponding to the EEG position Fp2 of the international 10–20 electrode system chosen as the stimulation site of right lateral OFC. Seed-to-voxel 1st level FC maps across groups and Pre/Post conditions were generated in the CONN toolbox and a second-level paired *t* test was used to examine the TMS-induced changes between targets and the rest of the brain in three groups separately. To assess whether FC changes were associated with the degree of symptom improvement in each group, Pre *v.* Post treatment seed-to-voxel connectivity maps were used to perform a multiple linear regression with percent score changes of HRSD-24 ((Pre–Post)/Pre) to identify brain regions exhibiting most relevant FC changes associated with symptom improvement. Regression analyses were also used to identify baseline FC that exhibited significant correlation with improvements in HRSD scores, serving as potential brain connectivity biomarkers for rTMS responses. The hypothesis-driven whole-brain result for Pre- and Post-FC changes using paired *t* test was thresholded using a voxel level of *p* < 0.005 and a cluster-based FDR correction of *p* < 0.05. Regression analyses assessing the treatment outcome related FC changes of TMS effect were thresholded using a voxel level of *p* < 0.005 and a cluster-based FDR of *p* < 0.004 (Bonferroni correction; 0.05 divided by 12 comparisons in the regression analyses between three experimental groups and two ROIs at Pre and Post time points) as correction for multiple comparisons.

### Statistical analysis

The range and distributions of patient demographic and baseline clinical variables were compared across groups via ANOVA or Wilcoxon signed rank test for continuous variables, and Pearson *χ*2 tests or Fisher exact test for categorical variables. Cohen's kappa coefficient (*κ*) was calculated to assess the success of blinding. Planned analyses to assess the rapidity of response focused on the time point immediately after treatment (Post). A series of linear mixed-effects models were calculated for the continuous change in scores in modified intention-to-treat (mITT, including participants who gave informed consent, were randomized, and received at least one session of study intervention) and per protocol samples (including participants who finished the treatment and follow-ups without major protocol deviations). The mITT population was the primary population of interest. The models contained group (Dual/Single/Sham), time (Pre/Post/1-week/4-week), and group and time interaction as fixed factors, and participant as random effect. Generalized estimating equation (GEE) was used to analyse binary longitudinal measurements (i.e. the response and remission rates over time), with a binomial distribution, a logit link, and an exchangeable correlation structure. All *p* values were 2-tailed, and statistical significance was defined as *p* < 0.05.

## Results

### Patients

A total of 102 patients were assessed with 75 patients (57 women) with a mean age 28.3 (s.d. 10.5) years and a mean baseline HRSD-24 of 45.7 (s.d. 10.7) randomly assigned to the three study arms (dual: *n* = 25; single: *n* = 25; and sham: *n* = 25). 68 participants (90.7%) were included for mITT analysis excluding 7 patients (who had inadvertently been found to be included in other studies) and 60 in the per-protocol (80.0%) analysis excluding 8 due to premature study termination (see online Supplementary Figure S1). Retention rates did not differ significantly between the three groups at any time point. 48.9% of the active groups (dual and single group) and 56.5% of the sham group (Sham group) correctly guessed their assigned treatment. There was no agreement between the participant's actual and perceived allocation (*κ* = 0.05, *p* = 0.67) indicating adequate participant blinding. Patient characteristics are shown in Table 1 for mITT sample and online supplementary Table S1 for per protocol sample.

**Table 1.** Baseline demographic and clinical characteristics by treatment arm

Characteristic	Dual	Single	Sham	p-value
Age at inclusion, mean (s.d.), y	27.7(9.5)	27.0(9.5)	29.6(11.2)	0.67
Gender, M/F	18/5	18/4	18/5	0.99
Education, mean (s.d.), y	12.6(2.0)	12.3(2.2)	12.3(2.3)	0.88
Duration since onset, mean (s.d.), y	4.4(6.3)	4.9(5.5)	2.3(1.8)	0.16
Baseline scores, mean (s.d.)				
HRSD-24	46.7(9.4)	44.4(10.4)	50.0(9.6)	0.16
HAMA	36.8(9.6)	33.8(7.4)	36.3(6.0)	0.38
Medications, no. (%)				
SSRIs	20(87.0)	19(86.4)	21(91.3)	0.90
Augmentation	22(95.7)	19(86.4)	23(100)	0.12
BDZs	20(87.0)	17(77.3)	22(95.7)	0.17

Abbreviations: HRSD-24, Hamilton Rating Scale for Depression 24-item version; HAMA, Hamilton Rating Scale for Anxiety; SSRIs, Selective Serotonin Reuptake Inhibitors; BDZs, Benzodiazepines.

### Clinical outcomes

**Figure 2.**
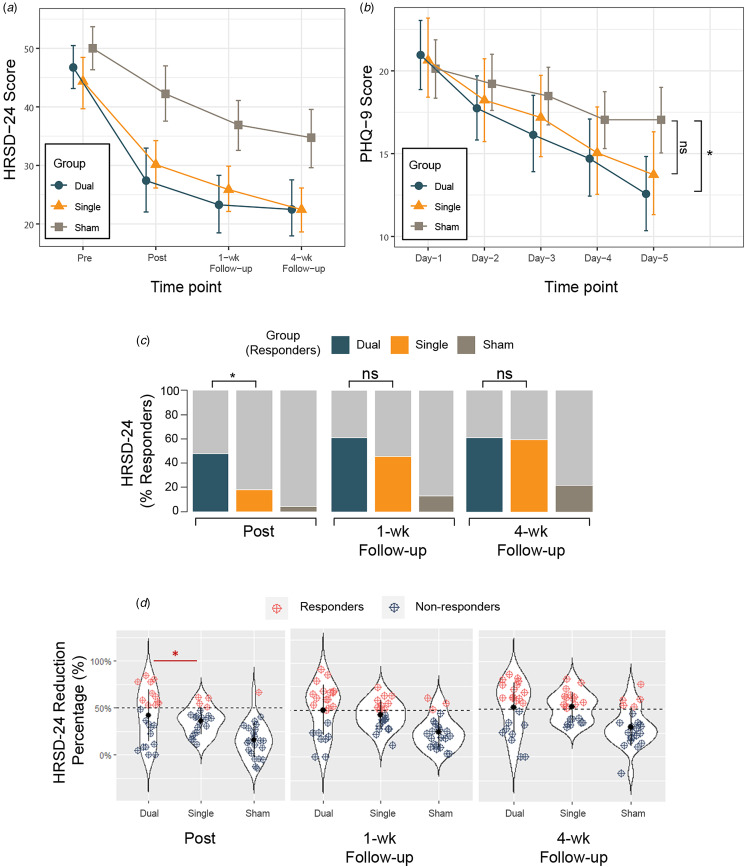
Changes in depression score over time in the modified intention-to-treat (mITT) population. (**A**) Mean score changes of the Hamilton Rating Scale of Depression (HRSD-24) at baseline (Pre), immediately after the completion of all treatment sessions (Post), 1 week (1-wk) and 4 weeks follow-up (4-wk) after the end of all treatment sessions. (**B**) The 9-question Patient Health Questionnaire (PHQ-9) daily changes during the TMS intervention (Day 1–5) demonstrating group by time effect. Significant difference was found on Day 5 showing lower PHQ-9 score in the Dual group compared to Sham with no significant difference between the Single and Sham. (**C**) Response (reduction of at least 50% from baseline score) rates of three groups over time based on HRSD-24 score change. The proportion of responders in the Dual group immediately after treatment completion was statistically higher than that in the Single group, with no significant differences at 1- and 4-week follow-up. (**D**) Percent changes in depression score over time in the modified intention-to-treat (mITT) population. Response defined as ⩾50% decrease from baseline total score of HRSD-24 (dashed black line). Blue dots indicate non-responders at different time points across groups and red dots indicate responders. The proportion of responders in the Dual group immediately after treatment completion was statistically higher than that in the Single group. Six more participants in the Single group turned into responders at 1 week follow-up and brought the response rate to 45.5% (Subj-1: 40.2% -> 51.0%; Subj-2: 39.2%->51.0%; Subj-3: 42.9%->55.1%; Subj-4: 40.0%->50.0%; Subj-5: 41.8%->52.7%; Subj-6: 46.9%->57.1%), with no significant differences found in response rate between Dual and Single group at 1- and 4-week follow-up. Abbreviations: HRSD-24, Hamilton Rating Scale of Depression, 24-item version; PHQ-9, 9-question Patient Health Questionnaire. The error bars represent 95% CIs; ns, not significant; *, *p*-value <0.05.

In the mITT sample, the primary analysis showed greater HRSD-24 reduction over time in the Dual and Single group than Sham (see Fig. 2A) reflected in a group by time interaction effect (*F*(6,204) = 4.30, *p* < 0.001) with moderate effect size (*η*p^2^ = 0.11; 95% CI 0.03–1.00) (Table 2). Planned post hoc analyses indicated a significant improvement in HRSD-24 assessed immediately after the final intervention (time point: Post) in the Dual compared to Sham (*p* < 0.001), and Single compared to Sham (*p* = 0.001), but no significant difference was found between the two treatment groups (Dual and Single group, *p* = 0.72). At 1-week and 4-week follow-up, results of HRSD-24 score changes yielded the same pattern of findings favoring the treatment (Dual/Single group) compared to Sham, with no difference between Dual and Single group.

**Table 2.** Study outcome measures according to treatment arm in the modified intention-to-treat (mITT) sample

Outcome measures	Dual		Single		Sham		LMMs or logistic regression	
	Participants (*N* = 23), No. (%)	Score, Mean (s.d.)	Participants (*N* = 22), No. (%)	Score, Mean (s.d.)	Participants (*N* = 23), No. (%)	Score, Mean (s.d.)	Group by time interaction	OR(95%CI): Dual *v.* Single
HRSD-24 score change								
−Baseline		46.7(9.5)		44.4(10.4)		50.0(9.6)	F(6,204) = 4.3***, *η*p2 = 0.11[0.03,0.18]	-
−Post		27.7(13.2)		30.1(10.5)		42.2(11.8)		
−1-week		23.3(12.2)		25.9(9.4)		36.9(11.2)		
−4-week		22.5(12.6)		22.5(9.2)		34.7(12.5)		
Clinical response (HRSD-24 score decreases by 50%)								
−Post	11(47.8)		4(18.2)		1(4.3)		-	4.6(1.1–18.8)*
−1-week	15(65.2)		10(45.5)		3(13.0)			2.2(0.7–7.4)
−4-week	14 (60.9)		13(59.1)		5(21.7)			1.1(0.3–3.5)
Clinical remission (HRSD-24 score⩽9)								
−Post	3(13.0)		0(0.0)		0(0.0)		-	ns
−1-week	2(8.7)		1(4.5)		0(0.0)			
−4-week	4(17.4)		2(9.1)		1(4.3)			
HAMA score change								
-Baseline		36.8 (9.6)		33.8(7.4)		36.3(6.0)	F(6,204) = 3.5**, *η*p2 = 0.09[0.01,0.15]	-
−Post		24.0(9.0)		22.7(5.5)		31.5(8.4)		
−1-week		20.5(10.5)		19.1(6.4)		28.6(8.7)		
−4-week		21.1(9.6)		19.3(8.6)		24.8(9.8)		
PHQ-9 score change								
−Day1		21.0(5.4)		20.6(5.7)		20.1(4.5)	F(8,272) = 2.4*, *η*p2 = 0.07[0.00,0.10]	-
−Day2		17.7(4.9)		18.2(6.4)		19.2(4.2)		
−Day3		16.1(5.9)		17.2(6.3)		18.5(4.5)		
−Day4		14.7(6.1)		15.0(6.7)		17.0(4.3)		
−Day5		12.6(6.0)		13.7(5.9)		17.0(5.3)		

Abbreviations: HRSD-24, Hamilton Rating Scale for Depression, 24 item version; HAMA, Hamilton Rating Scale for Anxiety; PHQ-9, 9-question Patient Health Questionnaire; LMMs, linear mixed models; *η*p2, partial eta squared; OR, odds ratio; ns, not significant. *, *p*-value <0.05; **, *p*-value <0.01; ***, *p*-value <0.001.

We then focused on the clinically relevant response (a reduction of at least 50% from HRSD-24 baseline) (Table 2; Figure 2C and 2D). In the mITT sample, a significant effect of group (GEE model, Wald = 13.4; *p* < 0.001) and time (Wald = 10.2; *p* = 0.001) was observed without significant interaction effect (Wald = 2.4; *p* = 0.12) on response rates. Planned analyses of the observed proportion of responders immediately after treatment (time point: Post) was significantly different across arms (47.8% v. 18.2% v. 4.3%, respectively; *χ*2 = 13.0, *p* = 0.002) with greater number of responders in the Dual compared to the Single (OR, 4.6; 95% CI 1.1–18.8; *p* = 0.03) and Dual *v.* Sham (OR, 18.9; 95% CI 2.15–165.9; *p* = 0.008) but not Single *v.* Sham (OR, 4.1; 95% CI 0.4–41.2; *p* = 0.23) group. Although between group differences remained at 1-week (65.2% v. 45.5% v. 13.0%) and 4-week follow-ups (60.9% v. 59.1% v. 21.7%) with more responders in the Dual relative to Single group, the inferential statistics do not support the hypothesis to reject a lack of difference at the population level. These results suggest a rapid onset of clinical response to TMS treatment in the Dual compared to Single group after the acute treatment phase. The per protocol analyses revealed similar results (see online supplementary Table S3).

The PHQ-9 was assessed over 5 days as a secondary outcome. We show time effect (*F*(4,272) = 40.4, *p* < 0.001) and group by time interaction (*F*(8,272) = 2.4, *p* = 0.02) but no group effect (*F*(2,68) = 1.1, *p* = 0.33) (Table 2). Between group differences on day 5 (Fig. 2B) showed PHQ-9 scores 4.5 points lower (95% CI −8.3 to 0.6; *p* = 0.02) in the Dual compared to Sham with no differences between Single and Sham (95% CI, −0.6 to 7.2; *p* = 0.11), with similar per protocol findings. Thus, both HRSD-24 response rate and PHQ-9 score highlight a rapid improvement in the Dual group with onset showed immediately after treatment completion on day 5.

### Tolerability and safety

Five different adverse events (AEs) were reported in the mITT sample (online supplementary Table S2). Significantly higher local pain at the target site in Dual *v.* Single and Sham was reported (47.8% v. 9.1% v. 0%, respectively; *p* < 0.001 Fisher's exact test). Thirteen (19.1%) patients reported mild to moderate transient headache without significant group difference. All events were considered potentially related to the TMS intervention itself and were limited to previously described adverse reactions, especially in the accelerated protocols (Chen, Hudaib, Hoy, & Fitzgerald, 2020).

### Overall post/pre-treatment connectivity changes

**Figure 3.**
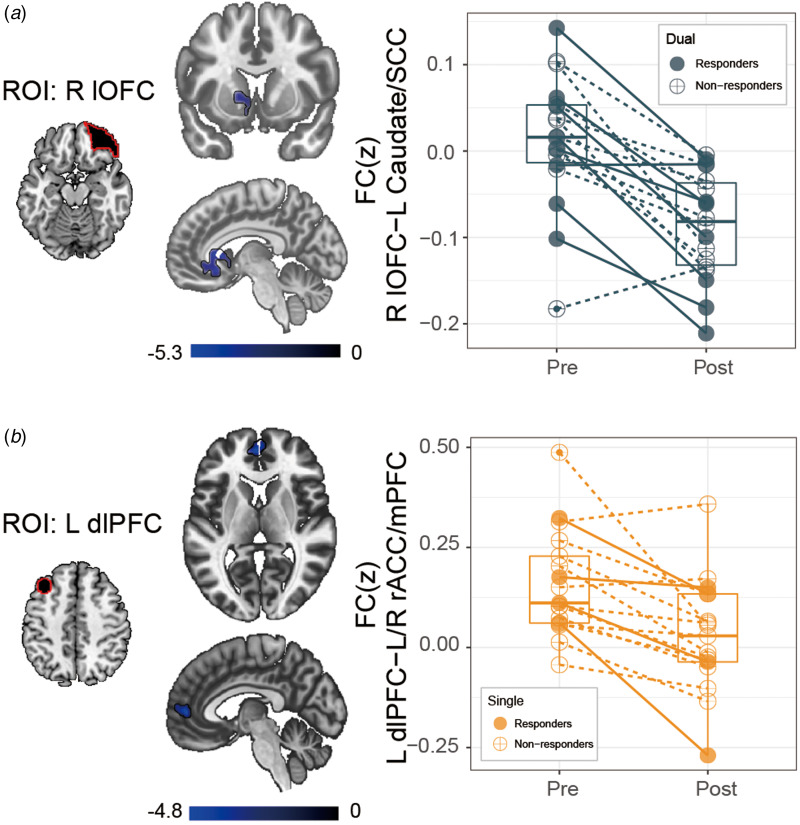
Baseline to post-treatment resting-state functional connectivity changes (Post > Pre) in the Dual, Single and Sham group. Values of the changes in functional connectivity (FC) were Fisher's *z* transformed (FC(z)). (**A**) In the Dual group, the right lateral orbitofrontal cortex (OFC) seed showed decreased connectivity after treatment with left subcallosal cingulate (SCC) (extending into left ventral striatum and caudate). (**B**) In the Single group, the left dorsolateral prefrontal cortex (dlPFC) seed showed decreased connectivity with the rostral anterior cingulate/medial PFC (rACC/mPFC). Scatter plots depicting the relationships between pre/post-treatment changes in FC are displayed for additional visualization of relationships. Blue solid dots indicate subjects from the Dual group that were clinical responders to the TMS intervention (percent reduction of HRSD-24⩾50%), while blue open dots indicate dual subjects ranked as non-responders to the TMS intervention at the completion of all treatment sessions (time point: Post); orange solid dots indicate subjects from the Single group that clinically responded to the TMS intervention, while orange open dots indicate subjects ranked as non-responders (time point: Post). Abbreviations: HRSD-24, Hamilton Rating Scale of Depression, 24 item version.

Resting state fMRI analyses focused on seed to whole brain FC with two ROIs located in the cortical targets of stimulation (right lateral OFC and left dlPFC). In the Dual group, there was a significant post-TMS decrease in FC between the right lateral OFC seed and left SCC extending into left ventral striatum and caudate (FDR *p* < 0.05, Fig. 3A) and a significant increase with left precentral gyrus (online supplementary Table S4). Left dlPFC seed also showed decreased FC with caudate post-treatment, along with FC changes in the right superior frontal, left occipital, and precentral gyrus (online supplementary Table S4). In the Single group, the left dlPFC seed exhibited decreased FC with rostral anterior cingulate/medial PFC (rACC/mPFC) (FDR *p* < 0.05, Fig. 3B). The lateral OFC seed showed significant FC changes with left inferior temporal gyrus (online supplementary Table S4). No significant change was found in the Sham group.

Thus, in keeping with our hypotheses, the Dual group showed post-treatment effects of decreased connectivity between lateral OFC and SCC and ventral striatum whereas the Single group showed decreased connectivity between the left dlPFC and mPFC including rACC.

### Correlates of clinical improvement: seed-to-voxel connectivity changes

**Figure 4.**
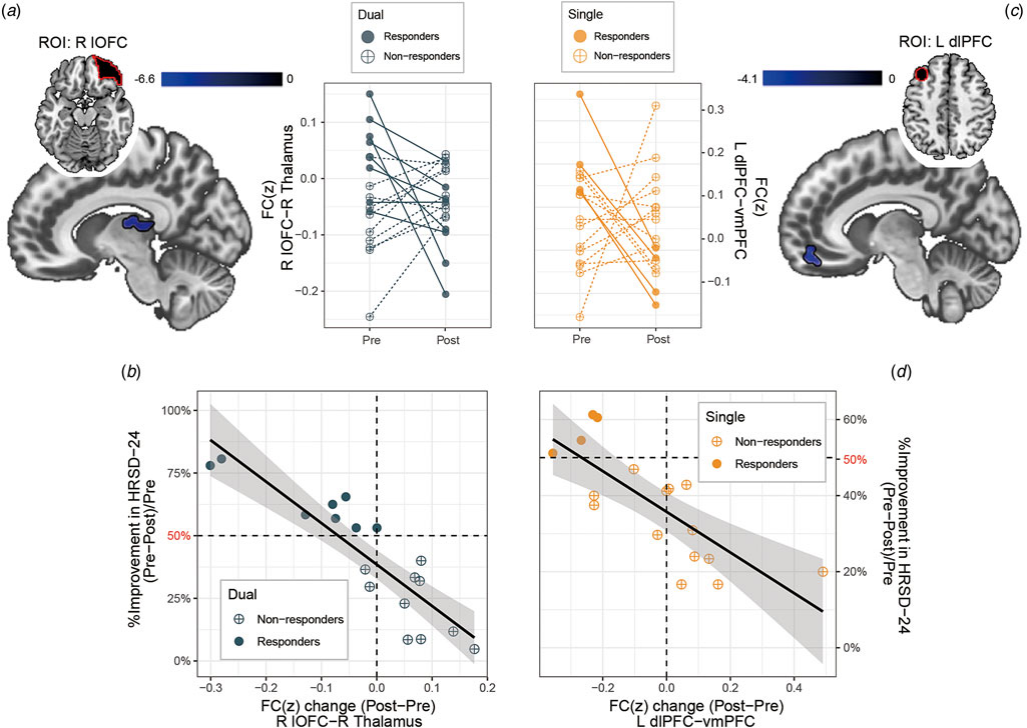
Treatment outcome related changes in functional connectivity. Treatment outcome (percent improvement in Hamilton Rating Scale of Depression (HRSD-24) after finishing all treatment sessions) related changes in intrinsic connectivity were found in the functional coupling of the right lateral orbitofrontal cortex (seed: R lOFC) – right thalamus in the Dual group, and the left dorsolateral prefrontal cortex (seed: L dlPFC) – ventromedial prefrontal cortex (vmPFC) in the Single group. Values of the changes in functional connectivity (FC) were Fisher's *z* transformed (FC(z)). (**A**) Changes of R lOFC-R thalamus FC negatively correlated with changes of HRSD-24 scores in subjects from the Dual group. On the bottom left, responders (percent reduction of HRSD-24⩾50% at the end of treatment) (blue solid dot) showed decreased FC with treatment (time point: Post), while non-responders (blue open dot) exhibited increased or stable FC with treatment. (**B**) Changes of L dlPFC-vmPFC FC negatively correlated with changes of HRSD-24 scores in subjects from the Single group. On the bottom right, responders (orange solid dot) showed decreased FC compared to baseline FC status, while non-responders (orange open dot) showed increased or stable FC after treatment. The solid black line is the linear regression with shaded area depicting the 95% confidence interval. Abbreviations: HRSD-24, Hamilton Rating Scale of Depression, 24 item version.

We then analysed clinical correlates using the %reduction in depression scores as a regressor to ask if TMS-induced FC changes correlate with symptom improvement (online supplementary Table S5). Larger reductions in HRSD-24 (Post>Pre) in the Dual group were associated with greater decreases in FC between the right lateral OFC seed and right lateral thalamus (FDR *p* < 0.004 Bonferroni corrected, Fig. 4A and 4B). In the Single group, greater depression improvement was associated with greater decreases in FC between dlPFC and ventromedial prefrontal cortex (vmPFC) (FDR *p* < 0.004 Bonferroni corrected, Fig. 4C and 4D). The Sham group had no significant findings.

### Baseline predictors of symptom improvement

**Figure 5.**
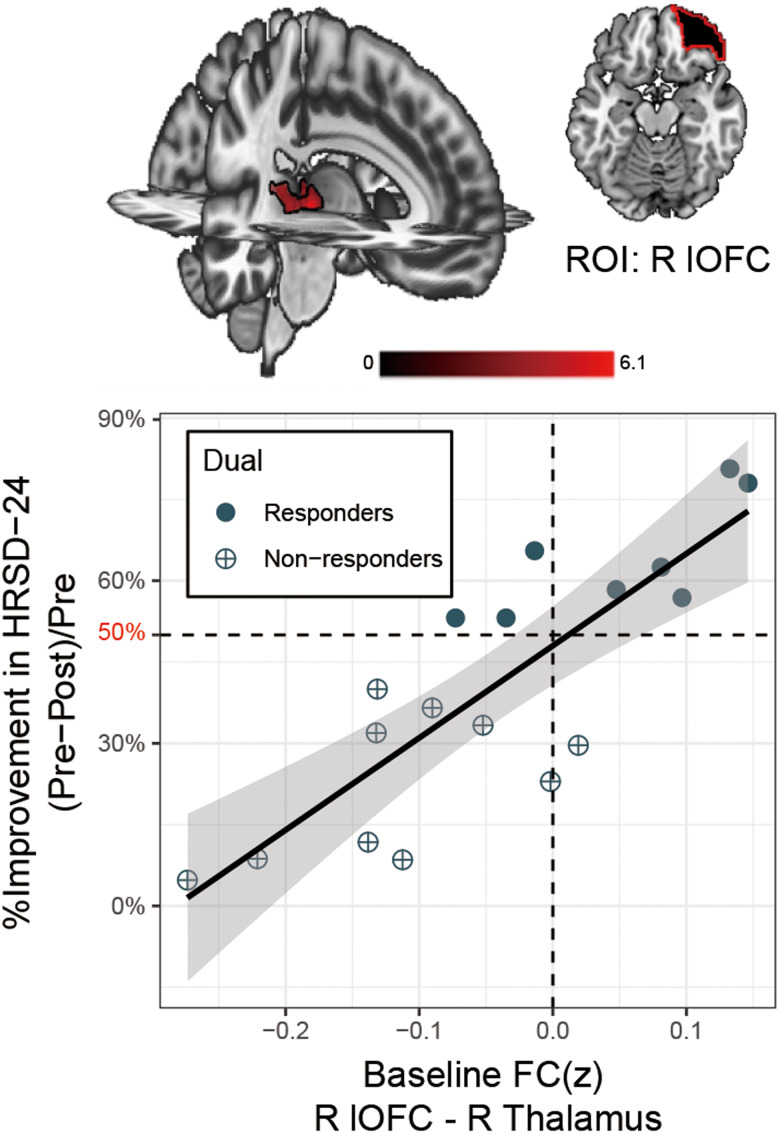
Neuroimaging predictors of treatment response. Responders were defined with ⩾50% improvements in Hamilton Rating Scale of Depression (HRSD-24) after the final session of TMS treatment. Baseline functional connectivity (FC) of the right lateral orbitofrontal cortex (R lOFC) seed to the right thalamus predicts subsequent treatment response to TMS in subjects allocated to the Dual group. Percent improvement in HRSD-24 is plotted on the vertical axis and baseline FC (Fisher's *z* transformed, FC(*z*)) of the right thalamus cluster to lOFC seed on the horizontal axis. The solid black line is the linear regression with shaded area depicting the 95% confidence interval. Improvement in HRSD-24 correlated with higher baseline FC between lOFC stimulation site and thalamus. Abbreviations: HRSD-24, Hamilton Rating Scale of Depression.

We then examined the relationship between baseline seed to whole brain FC relative to TMS clinical improvement (HRSD-24 %reduction after treatment completion) to assess predictors of outcome (online supplementary Table S6). In the Dual group, increased pre-treatment lateral OFC FC with the left mediodorsal thalamus predicted subsequent HRSD-24 improvement (FDR *p* < 0.004 Bonferroni corrected, Fig. 5), which is in accordance with the reported anatomical interconnection with the lateral OFC (Klein et al. 2010; Ray & Price, 1993). The Single group did not show significant findings (online supplementary Table S6).

## Discussion

To our knowledge, the present study is the first to examine the efficacy of a novel accelerated dual-site TMS protocol (Cambridge-Mind Fudan Accelerated Sequential TMS (CaM-FAST)) for the treatment of TRD combining OFC and dlPFC targets compared to an accelerated active control and sham. The dual-site stimulation protocol was safe with no severe adverse events reported among participants across all treatment sessions. We note that OFC stimulation can be particularly uncomfortable with higher local pain reported at the OFC target site in the Dual group (47.8%). However, the dual-site protocol was generally tolerated with reasonable dropout rate similar to the Single and Sham group. We instituted several methodological approaches to address the local discomfort of OFC stimulation including the intensity titration approach (70–100% rMT on the first two sessions and scaled up to 100% from the third session), which we adopted to ease the shock and pain resulting from the contraction of periocular muscles following the delivery of TMS pulses over OFC. This graded approach has been previously used in targeting the vmPFC (McCalley et al. 2023). Furthermore, we implemented the cTBS protocol, which exhibited advantages compared to low-frequency rTMS in terms of the shorter duration and was better received by patients, as indicated by feedback obtained during the piloting phase, particularly in minimizing local discomfort around the OFC area.

At the end of all sessions (time point: Post) and during follow-up (time point: 1-week and 4-week follow-up), significant reductions in HRSD scores were found in both the Dual (OFC- dlPFC) and the Single (sham OFC – dlPFC) group compared to Sham (sham OFC – sham dlPFC). However, we failed to show superiority of the novel dual-site protocol over the single-site stimulation in terms of the overall score change rated using the HRSD scale. As for secondary endpoints, a significantly higher rate of clinical response (reduction of at least 50% from baseline HRSD-24) was found in the Dual group immediately following the end of all sessions (time point: Post) compared to the Single group, along with a greater improvement on the self-rated PHQ-9 score on Day 5 in the Dual compared to the Sham group. We highlight this rapidity of the treatment effect with a larger proportion of clinical responders following the application of dual-site protocol, which may be particularly relevant to alleviate suicidal ideation, or severe distress or facilitate rapid discharge from hospital.

This novel protocol was predicated on several principles. First, we utilized an accelerated rTMS design at a higher frequency of 20 Hz targeting the left dlPFC. The significant reduction of HRSD score in the Single group adds to the existing literature supporting the efficacy of accelerated 20 Hz stimulation *v.* sham, whereas a prior crossover trial using accelerated 20 Hz rTMS (*n* = 20) did not demonstrate distinct effects on symptom improvements *v.* sham (Baeken et al. 2013). Despite variations in treatment acceleration (4 *v.* 5 sessions/day), target localization methods (EEG F3 *v.* anatomical landmarks of MFG) and total pulse counts (36 000 *v.* 31 200) that could affect treatment outcome, the current protocol features a longer ISI (50–60 min *v.* 15 min). This difference could potentially contribute to the overall efficacy of the present accelerated protocol (Downar, 2017), supported by evidence indicating more robust effects on synaptic plasticity with stimulation delivered at longer ISI (60–90 min) (Cao & Harris, 2014; Huang & Kandel, 1994; Kramar et al. 2012; Smolen, Zhang, & Byrne, 2016; Tse et al. 2018). Although the optimal ISI for therapeutic HF rTMS has not yet been systematically determined, recent accelerated designs, such as the SNT protocol, often lean towards selecting longer ISIs (Cole et al. 2020, 2022; Schulze et al. 2018).

Second, MDD is associated with heterogeneity reflected in multiple subtypes and networks. Here we target two different networks implicated in MDD, by additionally inhibiting the right lateral OFC commonly hyperactive in MDD (Drevets, 2007) and implicated in reward and loss valence representation relevant to MDD (Cheng et al. 2016). Our FC findings provide mechanistic evidence that the application of cTBS on the lateral OFC led to a decrease in the functional coupling between the OFC and the left SCC and ventral striatum, consistent with a recent study that compared the effects of iTBS and cTBS on the OFC network, revealing attenuated positive connectivity strength between the OFC and striatal ROIs following cTBS compared to iTBS (Price et al. 2023). In addition, we showed that the lateral OFC – thalamic FC correlated with improvement in depressive symptoms. Anatomically, the fiber tract that reciprocally interconnects OFC and thalamus is part of the inferior thalamic peduncle (ITP), which is dysregulated in MDD (Amiri, Arbabi, Kazemi, Parvaresh-Rizi, & Mirbagheri, 2021; Drevets, 2000a; Hauptman, DeSalles, Espinoza, Sedrak, & Ishida, 2008) and has been targeted with deep brain stimulation for TRD (Jimenez et al. 2013; Lee et al. 2019). This further underscores the significant involvement of the OFC frontostriatal network in depression (Drevets, 2000b; Rolls, 2019).

In the single group, we found that the post-TMS effect was associated with decreased dlPFC-rACC/vmPFC functional coupling (from positive value to negative value, ‘anticorrelation’), which was especially relevant to treatment responders rather than non-responders. This observation aligns with the extensive body of work highlighting the crucial interplay between the default mode network (DMN) and the central executive network (CEN) in depression (Bertocci et al. 2023; Han, Kim, Bae, Renshaw, & Anderson, 2016; Ho et al. 2015; Shapero et al. 2019). Specifically, it is in line with findings from Liston et al., where dlPFC stimulation with TMS tended to induce anticorrelations, characterized by a shift from positive FC at baseline to negative FC following TMS within the functional coupling between the dlPFC (as the seed region) and the medial prefrontal areas of the DMN (Liston et al. 2014). Prior studies on correlates of treatment response also include those examining TMS-induced FC changes between the subgenual ACC area and the dlPFC. For instance, Baeken and colleagues collected resting-state images before and after the treatment in a randomized sham-controlled crossover HF-rTMS study with 20 unipolar TRD patients (Baeken et al. 2014). Their results were consistent with two key studies conducted by Fox et al. (Fox, Buckner, White, Greicius, & Pascual-Leone, 2012; Fox, Liu, & Pascual-Leone, 2013), linking clinical outcome with suppression of the sgACC via dlPFC stimulation, and a stronger rsFC anti-correlation between the sgACC and parts of the left superior medial prefrontal cortex to be indicative of a better efficacy (Baeken et al. 2014). In a subsequent study, Baeken et al. evaluated the clinical effects of accelerated intermittent theta burst stimulation (aiTBS, 5 daily sessions spread over 4 days) on sgACC, but were unable to detect a negative baseline sgACC functional connectivity with the left dlPFC, or a subsequent reversal in association with a favorable clinical outcome (Baeken, Duprat, Wu, De Raedt, & van Heeringen, 2017). Instead, they found a strengthened sgACC–medial OFC FC accompanied by a decrease in feelings of hopelessness following TMS. Our imaging findings concerning the Single group, which corresponds to the dlPFC protocol (HF-TMS/iTBS) utilized in Baeken et al. (Baeken et al. 2014, 2017), did not align with their results. Nevertheless, we note that both studies have used sgACC as their seed region for FC analyses while ours were obtained using two cortical targets (i.e. OFC and dlPFC) as seed regions. As a result, the probable explanation for this discrepancy, notwithstanding methodological disparities, likely resides in the variance of seed region selection rather than disparate outcomes.

Moreover, we leveraged the observation that the state of network – which can be modulated – can potentially facilitate or inhibit subsequent effects of TMS. We theorize that there may be a ‘priming’ effect via the inhibition of the direct anatomical connection of right OFC to SCC prior to the standard HF stimulation of the left dlPFC. The concept of ‘priming’ or ‘preconditioning’ is potentially intriguing and plausible based on the physiological observations in MEP studies (Iyer, Schleper, & Wassermann, 2003). Experiments on healthy adults demonstrated that pairing of different protocols (cTBS -> iTBS or iTBS -> cTBS) showed a stronger effect on enhancing or inhibiting the MEP at the stimulated site than the use of testing protocol alone (Doeltgen & Ridding, 2011; Murakami, Muller-Dahlhaus, Lu, & Ziemann, 2012; Opie, Vosnakis, Ridding, Ziemann, & Semmler, 2017). As our result showed a decreased functional coupling between lateral OFC and SCC/ventral striatum following inhibitory TMS over lateral OFC along with known direct anatomical connection between the two regions, we speculate that the subsequent HF TMS over left dlPFC may be able to further enhance the putative excitatory impact of dlPFC rTMS on the SCC. Nonetheless, the current absence of findings demonstrating FC changes between the dlPFC and SCC may suggest that this hypothesis, although mechanistically plausible, requires further study. Additional research to clarify this putative ‘priming’ or state-related effect in TMS enhancement is needed.

Besides the relatively small sample size which requires further replication, several limitations of this pilot study should be noted. First, we did not include a fourth active arm stimulating the OFC site alone. Future study might consider having a separate arm dedicated to examining the isolated effect of OFC stimulation, along with the utilization of clinical scales or customized tasks to assess the impact of OFC stimulation on both clinical and psychological functions. Furthermore, we cannot exclude that the higher acute response rate in the Dual group may also related to the dose disparity (Theleritis et al. 2017), as the Dual group received pulses bilaterally, with 12 000 more pulses (25% more) received on the right lateral OFC compared to the Single group. Studies delivering higher doses (pulses/day) for longer treatment durations were generally believed more effective than those using lower doses in shorter protocols (Gershon, Dannon, & Grunhaus, 2003). The unequal number of pulses may have influenced the efficacy of different treatment arms, potentially leading to a bias toward a higher likelihood of response in the dual-site stimulation group. An alternative explanation of the disparities in treatment outcome is that the design of the dual-site stimulation could be considered as having doubled the TMS sessions per day. By pairing the OFC target with the dlPFC, the Dual group received twice the number of treatments (4 sessions/day -> 8 sessions/day) compared to the Single group. However, it is noteworthy that both the Single and Sham groups also received sham treatment sessions. Therefore, when factoring in sham sessions, all groups – Dual, Single, and Sham – received eight sessions per day. We believe that the discrepancy lies in whether the pulses administered in each session were real or sham. Future research should balance dosing strategy in treatment groups, although this can be challenging when applying stimulation at different targets or frequencies.

We also observed relatively lower rate of response (4/22) immediately following the end of all sessions in the Single group who received dlPFC stimulation. However, we note that a subgroup of six participants in the Single group exhibited ongoing improvement after completing all sessions, leading to a total response rate of 45.5% (10/22) at the 1-week follow-up (Fig. 2D). This delayed/continued response or improvement following rTMS treatment, though not statistically significant, is not uncommon with previous studies reporting similar trend of trajectories, even in groups receiving sham stimulation. For the sham conditions, besides potential TMS-induced modulative effect in the vertex area (see online supplementary results), this delayed and moderate improvement in symptoms may be related to several factors including continued medication through the course of TMS treatment, placebo effect (Razza et al. 2018), constant attention received by participants during the course of TMS treatment (Baeken et al. 2013), and spontaneous improvement (Cuijpers, Stringaris, & Wolpert, 2020; Whiteford et al. 2013). This last feature is of particular interest in the present cohort, given that the majority of recruited participants were predominantly female (averaging 79.4%) and generally fell within a younger age range (with an average age of 28.1 years), a period in which spontaneous improvement or remission commonly occurs (Thapar, Eyre, Patel, & Brent, 2022; Whiteford et al. 2013). During real TMS treatment, delayed clinical effect was found in a cohort studied by Duprat et al., with a response rate of 28% observed at the conclusion of the two-week TMS procedure, and 38% when evaluated two weeks after completing the sham-controlled protocol (Duprat et al. 2016). Note that this effect was only evident in the group that received actual stimulation in the first week, and the study with a crossover design was unable to examine whether it remained consistent in the group receiving sham stimulation first (Duprat et al. 2016). In another study comparing accelerated iTBS protocol to 4 weeks of standard daily rTMS, a continued improvement 4 weeks after the completion of treatment was observed among participants receiving accelerated protocol assessed using the Quick Inventory of Depressive Symptoms – subject rated (QIDS-SR) scale (Fitzgerald, Chen, Richardson, Daskalakis, & Hoy, 2020a). In the present study, however, it is worth noting that the six participants from the Single group assessed following acute treatment phase were in fact partial responders during their first post-TMS assessment and have narrowly missed the 50% reduction threshold (Subj-1: 40.2% -> 51.0%; Subj-2: 39.2%->51.0%; Subj-3: 42.9%->55.1%; Subj-4: 40.0%->50.0%; Subj-5: 41.8%->52.7%; Subj-6: 46.9%->57.1%; see Fig. 2D). Thus the ‘catching up’ of response rate in the Single group at 1-week follow-up assessment was not as abrupt as it first appeared. Taken together, the difference we found in the response rate at acute phase, and PHQ-9 differences between the Dual and Sham but not between Single and Sham at the end of 5-day treatment both indicates a potentially faster therapeutic effect possibly through targeting multiple networks. The use of higher doses or a longer protocol may further enhance the rapidity of onset and degree of improvement of findings perhaps distinguishing further from the active control.

The observed treatment outcome might also be impacted by the comorbid anxiety identified in this cohort. In our sample, 86.8% of patients were severe inpatients and were on stable doses of benzodiazepines for comorbid anxiety. In TMS studies, benzodiazepine use and concomitant anxiety are not strictly excluded given its common comorbidity; however, benzodiazepines have been suggested to have a potential adverse influence over the therapeutic effect of TMS (Deppe et al. 2021; Fitzgerald, Daskalakis, & Hoy, 2020b; Kaster et al. 2019). A better response rate might also have been achieved with the application of neuronavigation, particularly with TMS protocols targeting a single site. The use of neuronavigation enhances precision when employing standard protocols but can be costly and time-consuming along with limited feasibility for accurate targeting in accelerated protocols with multiple sessions in a single day (Fitzgerald, 2021; Fleischmann, Kohn, Trankner, Brandt, & Schmidt, 2020). Our dual-target accelerated protocol, designed in the context of these limitations, may be conceived as a strength in attempting to define novel means of optimizing treatment efficacy without the use of a neuronavigator and leading to a lower cost clinical generalizability.

In summary, we highlight a novel rTMS protocol for TRD using an accelerated dual-target method combining the classical dlPFC target, and lateral OFC which is implicated in the non-reward network. We demonstrate rapid clinical onset in response to dual-site stimulation consistent with a real-world clinical setting using an EEG 10–20 system guided accelerated approach. We emphasize the critical role of the OFC corticostriatal-thalamic network implicated in non-reward mechanisms underlying depression in predicting and tracking depression improvement. Further larger scale studies are indicated to confirm our findings and demonstrate the role of OFC connectivity as a predictive biomarker. Our findings have implications for novel designs of TMS protocols and implications for TRD management.

## Supporting information

Cui et al. supplementary material Cui et al. supplementary material

## Supplementary material

The supplementary material for this article can be found at https://doi.org/10.1017/S0033291724002289.
