# Supplementary material for: A novel dual-site OFC-dlPFC accelerated repetitive transcranial magnetic stimulation for depression: a pilot randomized controlled study
Source: Psychol Med. 2024 Oct 23;54(14):3849–62. doi: 10.1017/S0033291724002289 (PMC11578911; doi:10.1017/S0033291724002289)
Supplement: Cui et al. supplementary material [file S0033291724002289sup001.docx]

**Supplementary Information Appendix for “****A novel dual-site accelerated network priming repetitive transcranial magnetic stimulation for depression: a pilot randomized controlled study”: SI Materials and Methods, Results, Tables, and Figures**

**SI Materials and Methods**

*Eligibility Criteria*

The following criteria apply to those patients entering directly into the study. A patient may be included if all the following statements are satisfied.

1. Male or female, must be 18 to 55 years of age at time of screening with no previous experience of TMS treatment.
2. A primary diagnosis of major depressive disorder (MDD) without psychotic features as defined by DSM-V and international classification of disease (ICD)-10 criteria for a depressive episode, based on a structured interview (Mini International Neuropsychiatric Inventory.
3. Lack of antidepressant response or intolerance (via medical or pharmacy records) to a minimum of 2 but less than 4 adequate oral antidepressant treatment medications of adequate dose and duration in the current episode (maximum duration of current episode cannot exceed 5 years).
4. Hamilton Rating Scale of Depression, 24-item version (HRSD-24) (31) total score of 21 or more at the time of screening.
5. Medication free or current antidepressant or psychotropic medication regimen stable for at least 6 weeks before study entry with no expected change in medication during study.
6. Informed consent form must be signed indicating that the participant understands the purpose of and procedures required for the study and is willing to participate in the study.

The following exclusion criteria apply to those subjects entering directly into the study.

1. Participant has a current or prior DSM-IV-TR diagnosis of a major psychiatric disorder (e.g., substance use disorder, psychosis, bipolar disorder, anorexia, obsessive compulsive disorder) or MDD with psychotic features, bipolar or related disorders (confirmed by the MINI).
2. Acute suicidal or violent behaviour or history of suicide attempt within the last 4 weeks.
3. History of failure, or utilization during the current episode of Electroconvulsive Therapy (ECT) or repetitive Transcranial Magnetic Stimulation (rTMS); history of use at any time of Vagus Nerve Stimulation (VNS) or Deep Brain Stimulation (DBS).
4. Other severe or unstable medical condition ((e.g., major surgery or stroke) or neurological diseases (e.g., Parkinson’ disease) or evidence of cognitive impairments that could interfere with the conduct of the current study, or pose any unacceptable risk to the participant.
5. Presence of a specific contraindication for TMS/MRI (e.g., history of seizures, pacemaker, or metallic implant).
6. Use of psychotropic medications except for anti-depressant and sedative hypnotic medications.
7. Participant who is pregnant, breast-feeding, or planning to become pregnant while enrolled in this study.
8. History (within 1 year before the start of the screening) or current usage of an investigational drug or investigational medical device, or is currently enrolled in other investigational interventional studies.
9. Unable to comply with study visit schedule and timeline.

*Transcranial magnetic stimulation*

Using computer-generated randomization sequences, patients were 1:1:1 assigned to receive 1 of the 3 following treatments: (i) active continuous theta-burst stimulation (cTBS) targeting right lateral OFC (lOFC) followed by active 20Hz rTMS targeting left dorsal lateral prefrontal cortex (DLPFC) (Dual group); (ii) sham cTBS right lOFC followed by active rTMS left DLPFC (Single group), and (iii) sham cTBS right lOFC followed by sham rTMS left DLPFC (Sham group). The rationale of mixing cTBS with 20Hz rTMS was based on the following consideration. The TMS stimulation over the right lOFC site causes face and periocular muscles to contract and stimulates cutaneous fibres, which often leads to local pain or discomfort. At the piloting stage we found that this discomfort interfered with successful completion of experiments, with much higher dropout rate in the dual stimulation group (real lOFC stimulation included) compared to the single group, especially among TMS naïve patients whom were generally unfamiliar with the periocular sensation. We then examined the patients’ tolerability of one single session (600 pulses) of cTBS or 1Hz rTMS both with inhibitory impact over the lOFC site and demonstrated that the shorter 3.5 minutes cTBS stimulation of OFC was more acceptable and preferred among patients. Thus, cTBS instead of 1Hz rTMS was selected as the stimulation protocol for the right lOFC site to shorten the duration of discomfort and potentially reduce the dropout rate.

For the comparison of efficacy between rTMS and TBS, a previous large scale RCT study has demonstrated the non-inferiority of intermittent TBS (iTBS) over 10 Hz rTMS (Blumberger et al., 2018), while HF-rTMS of the left DLPFC has established anti-depressant efficacy (Lefaucheur et al., 2020). Notably, iTBS participants receiving a much shorter period of therapeutic contact than 10 Hz rTMS participants during each session could indeed increase the treatment capacity of clinics offering TMS therapy. However, due to a lack of sham-controlled studies assessing the antidepressant effect of iTBS delivered unilaterally to the left DLPFC, and the varied designs of stimulation protocols of iTBS, the latest guideline does not provide recommendation for iTBS as treatment of depression (Chen, 2020; Lefaucheur et al., 2020). Although 20 Hz rTMS has been shown to be more effective than 10 Hz, no studies have compared 20 Hz with sham in a larger RCT, hence our consideration in selecting the 20 Hz rTMS protocol.

*Sham group*

Patients randomized to the sham group underwent sham TMS stimulation for both OFC and dlPFC targets. We conducted the study in an adjunctive fashion such that subjects also received their prescribed treatments which were optimized but ineffective (and remain unchanged through the course of the study), which allowed subjects to get the full benefits of hospitalization, medication, and other supports from their psychiatrists. Participants were also informed that they were free to withdraw their consent to participate in the study at any time during data collection without having to give an explanation. In addition, participants in the sham group were given the option of receiving standard TMS treatment after they were unblinded at the end of the study, provided that their suitability for TMS intervention was confirmed by their psychiatrists. As a result, participants in the sham group were not deprived of all active treatments and we were very careful not to have exposed them to any unnecessary harm or discomfort, especially during the pandemic.

*MRI data acquisition*

MRI scans were acquired in a GE MR450 1.5-T scanner with an 8-channel head coil (General Electric, Madison, WI, USA) at baseline and after the intervention (no more than 120 min following the completion of the last treatment session). We acquired anatomical images via a T1-weighted magnetization-prepared rapid gradient echo (MPRAGE) sequence that lasted 5 min and 40 s. MPRAGE parameters were: 190 axial slices, slice thickness = 0.9 mm, TR/TE = 5/2.012 ms, FOV = 240 x 192 mm2, matrix size 256 x 256, flip angle = 8⁰, inversion time = 725 ms. Resting state T2*-weighted BOLD contrast images were acquired using gradient echo planar imaging (EPI) sequence covering the whole-brain. In this EPI sequence we obtained axial 28 slices, 2.0 × 2.0 × 3.5 mm resolution, spacing = 0.5mm, TE = 40 ms, TR = 2500 ms, flip angle = 90°, FOV = 240 x 240 mm2, 300 whole brain volumes, interleaved acquisition (from bottom to top).

*Statistical Power*

Sample size was calculated to detect a small to moderate effect. Using a type I error rate of 5% and a power of 80%, a total of 57 patients would be needed to detect an effect size of 0.18 (Cohen’s f). Considering a percentage of 20% dropouts, the final sample size is 75 patients, 25 in each group. The sample size calculation was performed using the G*Power 3.1 (Faul et al., 2007).

*Resting State Connectivity Processing Pipeline*

Resting state imaging data were preprocessed and seed-based connectivity calculated on the individual level using the CONN toolbox version 20b (Whitfield-Gabrieli & Nieto-Castanon, 2012), which implemented within the MATLAB R2021b software environment. The default pre-processing pipeline was utilized, including functional realignment and unwarping, slice timing correction, outlier identification (problematic time points were determined using the Artifact Detection Tools embedded within), structural and function segmentation and normalization, and functional smoothing with a 6.0 mm FWHM Gaussian smoothing kernel. Quality control settings corresponded to the “Intermediate” setting (97th percentile), with head displacement thresholded at 0.9mm from the previous frame in x, y, or z direction, or images of which the global mean intensity is greater than 5 standard deviations from the mean image intensity for the entire scan. Additional quality control cut-off acquired no more than 10% (30 volumes) of the entire run to be censored from further analysis. Denoising steps to avoid spurious correlations caused by head motion and other signal changes unrelated to brain activity were performed afterwards using aCompCor method, including linear regression of potential confounding effects in the BOLD signal, and temporal band-pass filtering. Specifically, subject-level general linear models (GLM) regressors consist of noise components from white matter and cerebrospinal areas (within each 5 potential noise components were estimated), estimated subject-motion parameters (3 rotation and 3 translation parameters, plus another 6 parameters representing their first-order temporal derivatives), and single time point regressors corresponding to outlier time points to be removed from the analysis (“scrubbing”). The resulting residual BOLD time series were linear detrended and band-pass filtered (0.008Hz < f < 0.09Hz) to focus on low-frequency fluctuations. We then checked for group differences in mean motion and number of volumes remaining after censoring obtained from the CONN toolbox. There were no significant differences in the number of censored volumes (Dual: 4.9±13.0; Single: 8.1±10.1; Sham: 10.1±11.9; p = 0.39) or in absolute mean motion (Dual: 0.08 ± 0.03; Single: 0.09 ± 0.03; Sham: 0.1±0.06; p = 0.55).

*MRI Data Analysis: conjunction analysis*

We first conducted a conjunction analysis (Friston et al., 2005) at baseline including all participants to assess common areas functionally connected to the two TMS targets. For functional connectivity (FC) analysis, two target areas were defined as regions of interest (ROIs): left dorsal lateral prefrontal cortex (dlPFC, Montreal Neurological Institute [MNI] coordinates = -38, 25, 48; 10-mm radius sphere), corresponding to the EEG positions F3 of the international 10 –20 electrode system chosen as the stimulation site of left dlPFC (Herwig et al., 2003), and right anterior and lateral orbitofrontal cortex (lOFC), created following AAL3 map labels using WFU Pickatlas software, corresponding to the EEG position Fp2 of the international 10-20 electrode system chosen as the stimulation site of right lateral OFC.

**SI Results**

*Conjunction analyses*

At baseline we asked how common areas functionally connected to the two TMS targets (right lOFC and left dlPFC) might overlap. Conjunction analyses revealed overlaps in the target areas (left dlPFC and right lOFC), dorsal anterior cingulate/medial prefrontal cortex, bilateral angular cortex, and middle temporal gyrus between the two resting state FC maps seeded at left dlPFC and right lOFC (**Fig.S2**). In keeping with our rationale of direct anatomical connectivity between the lOFC and subcallosal cingulate, the lOFC resting state functional connectivity map at baseline showed connectivity with subcallosal cingulate but not the dlPFC map. We then established that randomization had not introduced any group differences at baseline and focused the analyses on the within group post/pre-treatment functional connectivity changes induced by different TMS protocols.

*Vertex stimulation: neural substrates underlying the clinical improvement in the sham group*

Results from the hypothesis-driven seed-based FC analysis, using the TMS target sites as seeds (R lOFC and L dlPFC), did not reveal significant findings elucidating the mild clinical improvement of the sham group post-TMS treatment. As an exploratory analysis, we examined whether vertex as a target produced significant pre- versus post-TMS FC changes. Similar to the FC analyses seeded in the OFC and dlPFC, we then evaluated whether any observed FC alterations with the vertex target might underlie the symptom improvement observed at the conclusion of all treatment sessions in the sham group. Using spheric ROI (r=10mm) seeded in the EEG-Cz location (MNI[-0.5,-24,80]), which corresponds to the anatomical location of the vertex (Scrivener & Reader, 2022), we found no significant pre- versus post FC changes with the vertex ROI at a threshold of voxel p<0.005 uncorrected, cluster-size p<0.05 (FDR correction). Without cluster size correction, we found increased FC between the vertex and the left middle frontal cortex, the right temporal pole, and the right angular gyrus (**Table S7**). This finding is not dissimilar with a previous study showing significant deactivation within the DMN areas, given the different significant threshold applied to the outcomes of the analyses (Jung et al., 2016).

Subsequently, we conducted an analysis of clinical correlates, employing the percentage reduction in depression scores after the treatment ((Pre-Post)/Pre) as a regressor to determine whether seed-based functional connectivity (FC) changes correlated with improvements in clinical depression. At a threshold of voxel p<0.005 uncorrected and cluster-size p<0.05 (FDR correction), no significant area was found showing clinical improvement related FC with the vertex seed. At a lower threshold with cluster-size uncorrected (p<0.05), greater symptom improvement in the sham group was found to be associated with greater reduction in the FC between the vertex seed and the vmPFC, subcallosal cingulate, and right precuneus (**Table S8**). This result demonstrated that vertex stimulation might induce a deactivation in the subgenual ACC at a non-significant level, that may potentially contribute to the clinical improvement showing in the sham group, alongside other factors discussed in the main text: (1) continued medication through the course of TMS treatment, (2) placebo effect (Razza et al., 2018), (3) constant attention received by participants during the course of TMS treatment (Baeken et al., 2013), and (4) spontaneous improvement (Cuijpers et al., 2020; Whiteford et al., 2013). However, cautious needs to be taken given the significance threshold applied to the FC results.

**References**

Blumberger, D. M., Vila-Rodriguez, F., Thorpe, K. E., Feffer, K., Noda, Y., Giacobbe, P.,…Downar, J. (2018). Effectiveness of theta burst versus high-frequency repetitive transcranial magnetic stimulation in patients with depression (THREE-D): a randomised non-inferiority trial. *Lancet*, *391*(10131), 1683-1692. https://doi.org/10.1016/S0140-6736(18)30295-2

Chen, R. (2020). Guideline on therapeutic use of repetitive transcranial magnetic stimulation: Useful but know the methods and limitations. *Clinical Neurophysiology*, *131*(2), 461-462. https://doi.org/10.1016/j.clinph.2019.11.020

Faul, F., Erdfelder, E., Lang, A. G., & Buchner, A. (2007). G*Power 3: a flexible statistical power analysis program for the social, behavioral, and biomedical sciences. *Behavior Research Methods*, *39*(2), 175-191. https://doi.org/10.3758/bf03193146

Friston, K. J., Penny, W. D., & Glaser, D. E. (2005). Conjunction revisited. *Neuroimage*, *25*(3), 661-667. https://doi.org/10.1016/j.neuroimage.2005.01.013

Herwig, U., Lampe, Y., Juengling, F. D., Wunderlich, A., Walter, H., Spitzer, M., & Schonfeldt-Lecuona, C. (2003). Add-on rTMS for treatment of depression: a pilot study using stereotaxic coil-navigation according to PET data. *Journal of Psychiatric Research*, *37*(4), 267-275. https://doi.org/10.1016/s0022-3956(03)00042-6

Jung, J., Bungert, A., Bowtell, R., & Jackson, S. R. (2016). Vertex Stimulation as a Control Site for Transcranial Magnetic Stimulation: A Concurrent TMS/fMRI Study. *Brain Stimulation*, *9*(1), 58-64. https://doi.org/10.1016/j.brs.2015.09.008

Lefaucheur, J. P., Aleman, A., Baeken, C., Benninger, D. H., Brunelin, J., Di Lazzaro, V.,…Ziemann, U. (2020). Evidence-based guidelines on the therapeutic use of repetitive transcranial magnetic stimulation (rTMS): An update (2014-2018). *Clinical Neurophysiology*, *131*(2), 474-528. https://doi.org/10.1016/j.clinph.2019.11.002

Scrivener, C. L., & Reader, A. T. (2022). Variability of EEG electrode positions and their underlying brain regions: visualizing gel artifacts from a simultaneous EEG-fMRI dataset. *Brain and Behavior*, *12*(2), e2476. https://doi.org/10.1002/brb3.2476

Whitfield-Gabrieli, S., & Nieto-Castanon, A. (2012). Conn: a functional connectivity toolbox for correlated and anticorrelated brain networks. *Brain Connectivity*, *2*(3), 125-141. https://doi.org/10.1089/brain.2012.0073

**SI Tables**

**Table S1. Baseline demographic and clinical characteristics by treatment arm in the per protocol sample.**

| Characteristic | Dual | Single | Sham | P value |
| --- | --- | --- | --- | --- |
| Age at inclusion, mean (SD), y | 27.6(9.6) | 26.3(8.6) | 29.5(11.3) | 0.57^a^ |
| Gender, M/F | 4/15 | 4/16 | 5/16 | 0.99^b^ |
| Education, mean (SD), y | 12.7(2.0) | 12.4(2.1) | 12.4(2.3) | 0.86^a^ |
| Duration since onset, mean (SD), y | 4.8(6.8) | 4.8(5.7) | 2.4(1.7) | 0.24^a^ |
| Baseline scores, mean (SD) | | | | |
| HRSD-24 | 47.9 (9.5) | 45.1(9.9) | 51.2(9.1) | 0.13^a^ |
| HAMA | 35.4 (9.4) | 34.3(7.6) | 36.8(6.0) | 0.57^a^ |
| Medications, No. (%) | | | | |
| SSRIs | 19(100.0) | 19(95.0) | 20(95.2) | 0.99^b^ |
| Augmentation | 19(100.0) | 17(85.0) | 21(100.0) | 0.06^b^ |
| BDZs | 19(100.0) | 16(80.0) | 20(95.2) | 0.07^b^ |

Abbreviations: HDRS-24, Hamilton Rating Scale of Depression, 24-item version; HAMA, Hamilton Rating Scale of Anxiety; BDZs, Benzodiazepines; SSRIs, Selective serotonin reuptake inhibitors.

^a^ One-way ANOVA.

^b^ Fisher’s exact test.

**Table S2. Full list of reported adverse events according to treatment arm in the modified intention-to-treat (mITT) sample.**

| Type of adverse event | Total Number (N=68) | Dual (N=23) | Single (N=22) | Sham (N=23) | p-value | Post-hoc | | |
| --- | --- | --- | --- | --- | --- | --- | --- | --- |
|  |  |  |  |  |  | **Dual vs Single** | **Dual vs Sham** | **Single vs Sham** |
|  |  |  |  |  |  | p-value | p-value | p-value |
| Headache, No. (%) | 13(19.1) | 5(21.7) | 5(22.7) | 3(13.0) | 0.74^a^ | - | - | - |
| Local pain at the stimulation site, No. (%) | 13(19.1) | 11(47.8) | 2(9.1) | 0(0) | <0.001^a^ | 0.02 | <0.001 | 0.23 |
| Dizziness, No. (%) | 9(13.2) | 4(17.4) | 3(13.6) | 2(8.7) | 0.76^a^ | - | - | - |
| Nausea, No. (%) | 4(5.9) | 3(13.0) | 1(4.5) | 0(0) | 0.21^a^ | - | - | - |
| Sleeping problem, No. (%) | 1(1.5) | 0(0.0) | 1(4.5) | 0(0) | 0.32^a^ | - | - | - |

^a^ Fisher’s exact test followed by pairwise comparisons if p<0.05, Bonferroni corrected

**Table S3. Study outcome measures according to treatment arm in the per protocol sample.**

| Outcome measures | Dual Group | | Single Group | | Sham Group | | LMMs or logistic regression | |
| --- | --- | --- | --- | --- | --- | --- | --- | --- |
|  | Participants (N=19),  No. (%) | Score,  Mean (SD) | Participants (N=20),  No. (%) | Score,  Mean (SD) | Participants (N=21),  No. (%) | Score,  Mean (SD) | Group by Time interaction | OR[95%CI]: Dual versus Single |
| HRSD-24 score change | | | | | | | | |
| -Baseline |  | 47.9(9.5) |  | 45.2(9.9) |  | 51.2(9.1) | F(6,180)=5.0***,  ηp2=0.14[0.04,0.22] | - |
| -Post |  | 27.8(13.2) |  | 30.8(10.4) |  | 43.7(11.3) |  |  |
| -1-week |  | 22.5(11.9) |  | 26.1(9.5) |  | 37.9(11.2) |  |  |
| -4-week |  | 21.5(12.4) |  | 22.4(9.2) |  | 35.5(12.8) |  |  |
| Clinical response (HRSD-24 score decreases by 50%) | | | | | | | | |
| -Post | 10(52.6) |  | 4(20.0) |  | 1(4.8) |  | - | 6.0[1.3-28.3]* |
| -1-week | 13(68.4) |  | 10(50.0) |  | 3(14.3) |  |  | 2.3[0.6-8.4] |
| -4-week | 12 (63.2) |  | 13(65.0) |  | 5(23.8) |  |  | 0.9[0.2-3.6] |
| Clinical remission (HRSD-24 score ≤9) | | | | | | | | |
| -Post | 3(15.8) |  | 0(0.0) |  | 0(0.0) |  | - | ns |
| -1-week | 2(10.5) |  | 1(4.5) |  | 0(0.0) |  |  |  |
| -4-week | 4(21.1) |  | 2(9.1) |  | 1(4.3) |  |  |  |
| HAMA score change | | | | | | | | |
| -Baseline |  | 35.4 (9.4) |  | 34.3(7.6) |  | 36.8(6.0) | F(6,180)=4.0***,  ηp2=0.12[0.02,0.19] | - |
| -Post |  | 21.8 (7.9) |  | 22.8(5.7) |  | 32.5(8.1) |  |  |
| -1-week |  | 18.3(9.4) |  | 18.8(6.6) |  | 29.3(8.8) |  |  |
| -4-week |  | 19.0(8.4) |  | 19.1(8.9) |  | 25.1(10.2) |  |  |
| PHQ-9 score change | | | | | | | | |
| -Day1 |  | 21.1(5.8) |  | 20.9(5.9) |  | 20.4(4.6) | F(8,240)=2.8**,  ηp2=0.09[0.01,0.13] |  |
| -Day2 |  | 17.4(5.1) |  | 18.4(6.7) |  | 19.7(4.1) |  |  |
| -Day3 |  | 15.6(6.0) |  | 17.2(6.5) |  | 18.7(4.6) |  |  |
| -Day4 |  | 14.3(5.5) |  | 15.0(7.0) |  | 17.0(4.5) |  |  |
| -Day5 |  | 11.5(5.3) |  | 13.4(6.1) |  | 17.1(5.5) |  |  |

Abbreviations: HRSD-24, Hamilton Rating Scale for Depression, 24-item version; HAMA, Hamilton Rating Scale for Anxiety; PHQ-9, 9-question Patient Health Questionnaire; LMMs, linear mixed models; ηp2, partial eta squared; OR, odds ratio; ns, not significant. *, p-value <0.05; **, p-value <0.01; ***, p-value <0.001.

**Table S4. Brain regions underlying significant TMS-induced changes (Post>Pre) in seed-to-voxel functional connectivity in each group (FDR p<.05).**

| Group | Brain Regions | L/R | Brodmann area (BA) | Cluster extent (K_E_) | Statistics | Effect size | Peak MNI coordinates  (x, y, z) | | |
| --- | --- | --- | --- | --- | --- | --- | --- | --- | --- |
| Seed: Right lateral orbitofrontal cortex (R lOFC) | | | | | | | | | |
| Dual | Subcallosal cingulate (SCC, extending into left ventral striatum and caudate) | L | 25 | 209 | -4.00 | -1.52 | -14 | 24 | 2 |
| - | Precentral gyrus (PreCG) | L | 4 | 366 | 3.60 | 1.11 | -38 | -18 | 56 |
| Seed: Left dorsal lateral prefrontal cortex (L dlPFC) | | | | | | | | | |
| Dual | Lateral occipital cortex | L | 19 | 312 | 4.53 | 1.68 | -22 | -84 | 32 |
| - | Superior frontal cortex (SFC) | R | 10 | 196 | -3.95 | -1.17 | 16 | 56 | 18 |
| - | Caudate (extending into ventral striatum and SCC) | R | - | 175 | -3.40 | -1.32 | 8 | 10 | 6 |
| - | Precentral gyrus (PreCG) | L | 5 | 138 | 3.86 | 1.51 | -6 | -30 | 50 |
| Seed: Right lateral orbitofrontal cortex (R lOFC) | | | | | | | | | |
| Single | Inferior temporal gyrus (ITG) | L | 20 | 191 | -4.95 | -1.58 | -46 | -12 | -38 |
| Seed: Left dorsal lateral prefrontal cortex (L dlPFC) | | | | | | | | | |
| Single | Rostral anterior cingulate (rACC)/medial prefrontal cortex (mPFC) | L/R | 10 | 406 | -4.73 | -1.07 | 8 | 48 | 10 |
| Seed: Right lateral orbitofrontal cortex (R lOFC) | | | | | | | | | |
| Sham | None |  |  |  |  |  |  |  |  |
| Seed: Left dorsal lateral prefrontal cortex (L dlPFC) | | | | | | | | | |
| Sham | None |  |  |  |  |  |  |  |  |

**Table S5. Brain regions underlying significant correlation between treatment outcome (percent improvement of HRSD-24) and TMS-induced changes (Post>Pre) in seed-to-voxel functional connectivity in each group (FDR p<.004, Bonferroni correction, 0.05 divided by 12 comparisons in the regression analyses between three experimental groups and two ROIs).**

| Group | Brain Regions | L/R | Brodmann area (BA) | Cluster extent (K_E_) | Statistics | Effect size (correlation coefficient) | Peak MNI coordinates  (x, y, z) | | |
| --- | --- | --- | --- | --- | --- | --- | --- | --- | --- |
| Seed: Right lateral orbitofrontal cortex (R lOFC) | | | | | | | | | |
| Dual | Thalamus (Thal_LP) | R | - | 454 | -7.58 | -0.88 | 6 | -18 | 12 |
| Seed: Left dorsal lateral prefrontal cortex (L dlPFC) | | | | | | | | | |
| Dual | None |  |  |  |  |  |  |  |  |
| Seed: Right lateral orbitofrontal cortex (R lOFC) | | | | | | | | | |
| Single | Angular gyrus | R | 39 | 321 | -6.33 | -0.85 | 40 | -54 | 30 |
| Seed: Left dorsal lateral prefrontal cortex (L dlPFC) | | | | | | | | | |
| Single | Ventral medial prefrontal cortex (vmPFC) | L/R | 10 | 176 | -4.64 | -0.77 | 2 | 46 | -28 |
| Seed: Right lateral orbitofrontal cortex (R lOFC) | | | | | | | | | |
| Sham | None |  |  |  |  |  |  |  |  |
| Seed: Left dorsal lateral prefrontal cortex (L dlPFC) | | | | | | | | | |
| Sham | None |  |  |  |  |  |  |  |  |

**Table S6. Brain regions** **underlying significant correlation between treatment outcome (percent improvement of HRSD-24) and baseline seed-to-voxel functional connectivity in each group (FDR p<.004, Bonferroni correction, 0.05 divided by 12 comparisons in the regression analyses between three experimental groups and two ROIs).**

| Group | Brain Regions | L/R | Brodmann area (BA) | Cluster extent (K_E_) | Statistics | Effect size (correlation coefficient) | Peak MNI coordinates  (x, y, z) | | |
| --- | --- | --- | --- | --- | --- | --- | --- | --- | --- |
| Seed: Right lateral orbitofrontal cortex (R lOFC) | | | | | | | | | |
| Dual | Thalamus (Thal_MD) | L | - | 521 | 6.65 | 0.80 | -6 | -24 | 0 |
| Seed: Left dorsal lateral prefrontal cortex (L dlPFC) | | | | | | | | | |
| Dual | None |  |  |  |  |  |  |  |  |
| Seed: Right lateral orbitofrontal cortex (R lOFC) | | | | | | | | | |
| Single | None |  |  |  |  |  |  |  |  |
| Seed: Left dorsal lateral prefrontal cortex (L dlPFC) | | | | | | | | | |
| Single | None |  |  |  |  |  |  |  |  |
| Seed: Right lateral orbitofrontal cortex (R lOFC) | | | | | | | | | |
| Sham | None |  |  |  |  |  |  |  |  |
| Seed: Left dorsal lateral prefrontal cortex (L dlPFC) | | | | | | | | | |
| Sham | Occipital fusiform gyrus | L | 37 | 508 | 6.83 | 0.85 | -34 | -46 | -14 |

**Table S7. Brain regions showing TMS-induced vertex-seeded functional connectivity changes (Post>Pre) in the sham group.**

| Group | Brain Regions | L/R | Brodmann area (BA) | Cluster extent (K_E_) | Statistics | Effect size | Peak MNI coordinates  (x, y, z) | | |
| --- | --- | --- | --- | --- | --- | --- | --- | --- | --- |
| Threshold: voxel p<0.005 uncorrected, cluster-size p<0.05 FDR-corrected | | | | | | | | | |
| Sham | None |  |  |  |  |  |  |  |  |
| Threshold: voxel p<0.005 uncorrected, cluster-size p<0.05 uncorrected | | | | | | | | | |
| Sham | Middle frontal cortex | L | 8 | 74 | - | - | -40 | 6 | 36 |
|  | Angular gyrus | R | 39 | 54 |  |  | 60 | -52 | 36 |
|  | Temporal pole | R | 6 | 71 | - | - | 52 | 2 | -28 |

**Table S8. Brain regions showing correlation between treatment outcome (percent improvement of HRSD-24 at the end of all sessions) and TMS-induced vertex-seeded functional connectivity changes (Post>Pre) in the sham group.**

| Group | Brain Regions | L/R | Brodmann area (BA) | Cluster extent (K_E_) | Statistics | Effect size | Peak MNI coordinates  (x, y, z) | | |
| --- | --- | --- | --- | --- | --- | --- | --- | --- | --- |
| Threshold: voxel p<0.005 uncorrected, cluster-size p<0.05 FDR-corrected | | | | | | | | | |
| Sham | None |  |  |  |  |  |  |  |  |
| Threshold: voxel p<0.005 uncorrected, cluster-size p<0.05 uncorrected | | | | | | | | | |
| Sham | Ventromedial prefrontal cortex | L/R | 11 | 103 | - | - | -4 | 40 | -22 |
|  | Subcallosal cingulate | R | 11 | 75 |  |  | 14 | 28 | -18 |
|  | Precuneus | R | 31 | 67 | - | - | 10 | -56 | 38 |

**SI Figures**

**
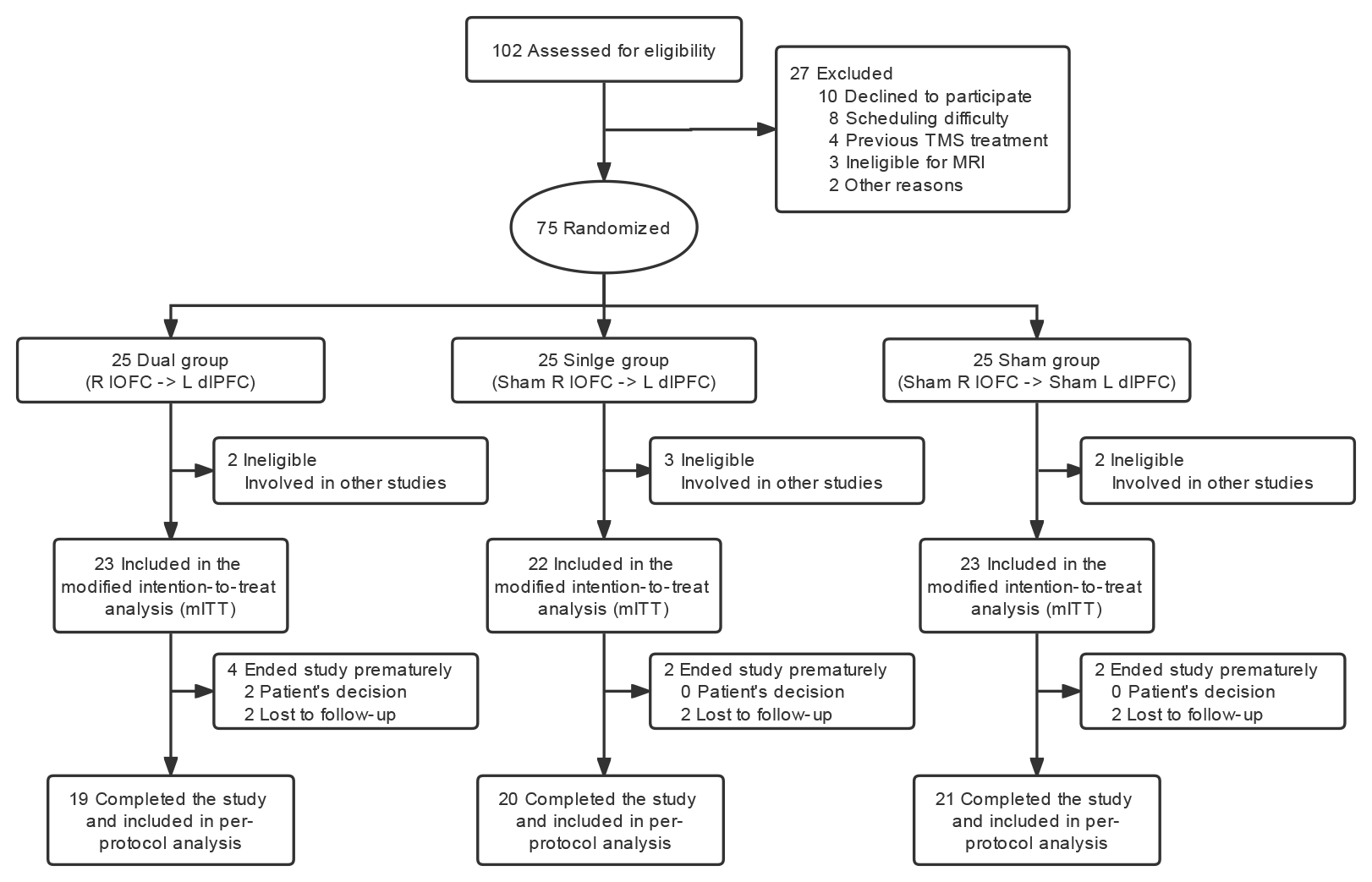
**

**Figure S1. CONSORT Flow Diagram.** A total of 102 patients were assessed for eligibility, and a total of 75 patients (57 women, 18 men) with a mean (SD) age of 28.3 (10.5) years and a mean (SD) baseline HRSD-24 score of 45.7 (10.7) were randomly assigned to the 3 study arms (Dual group: inhibitory cTBS right lOFC followed by excitatory 20Hz rTMS left dlPFC, n = 25; Single group: sham inhibitory cTBS right lOFC followed by excitatory 20Hz rTMS left dlPFC, n = 25; and Sham group: sham right lOFC followed by sham left dlPFC, n = 25). A total of 68 participants (mean [SD] age, 28.1 [10.0] years; HRSD-24 score at baseline, 47.1 [10.0]) were included for modified intention-to-treat (mITT) analyses, excluding 7 participants that later found to be inadvertently involved in other ongoing studies. 60 participants (mean [SD] age, 27.8 [9.9] years; HRSD-24 score at baseline, 48.6 [9.9]) completed the study (Dual group: n = 19; Single group: n = 20; and Sham group: n = 21) without major protocol violation. The analyses were conducted on both modified intent-to-treat (mITT) and per protocol bases with similar findings. Abbreviations: rTMS, repetitive transcranial magnetic stimulation; cTBS, continuous theta burst stimulation; lOFC, lateral orbitofrontal cortex; dlPFC, dorsal lateral prefrontal cortex.

**
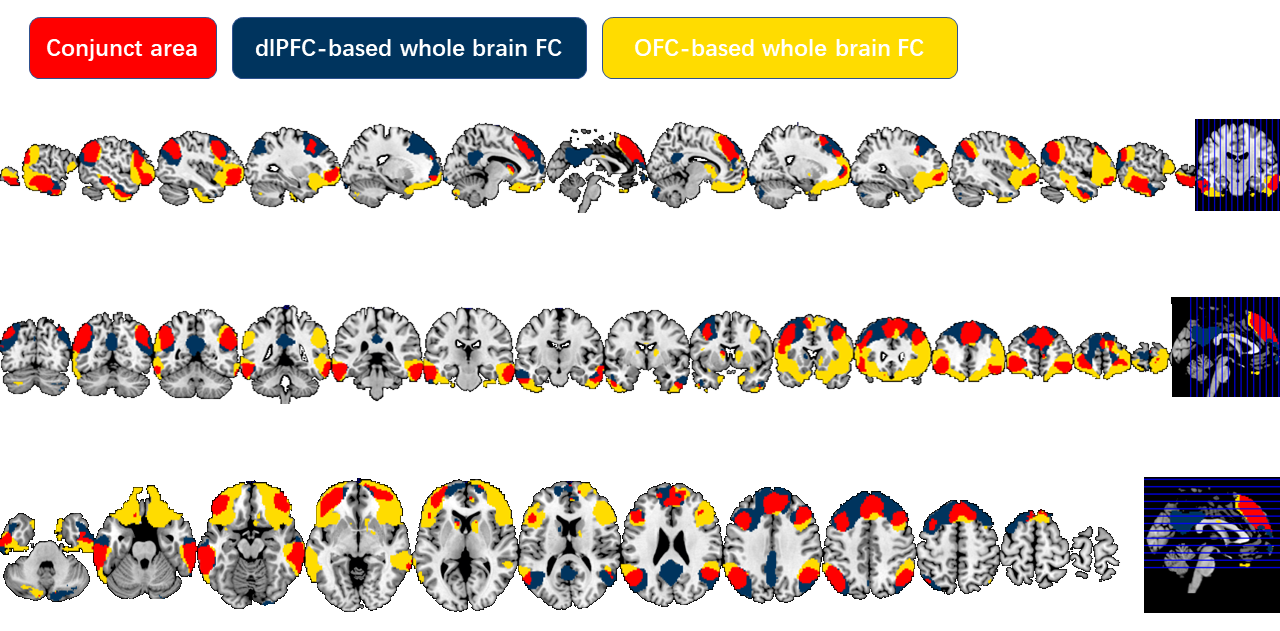
**

**Figure S2.** **Conjunction analysis result.** Seed-based resting state functional connectivity at baseline across left dorsal lateral prefrontal cortex (dlPFC, areas coloured in dark blue) and right lateral orbital frontal cortex (lOFC, areas coloured in yellow) reveals conjunct brain areas (red) comprising the dorsal anterior cingulate cortex (dACC)/medial PFC, lateral OFC, dlPFC, angular cortex and middle temporal gyrus.
